# Applying multidimensional computerized adaptive testing to the MSQOL-54: a simulation study

**DOI:** 10.1186/s12955-023-02152-8

**Published:** 2023-06-25

**Authors:** Andrea Giordano, Silvia Testa, Marta Bassi, Sabina Cilia, Antonio Bertolotto, Maria Esmeralda Quartuccio, Erika Pietrolongo, Monica Falautano, Monica Grobberio, Claudia Niccolai, Beatrice Allegri, Rosa Gemma Viterbo, Paolo Confalonieri, Ambra Mara Giovannetti, Eleonora Cocco, Maria Grazia Grasso, Alessandra Lugaresi, Elisa Ferriani, Ugo Nocentini, Mauro Zaffaroni, Alysha De Livera, George Jelinek, Alessandra Solari, Rosalba Rosato

**Affiliations:** 1 Unit of Neuroepidemiology, Fondazione IRRCS Istituto Neurologico Carlo Besta, Via Celoria 11, Milan, 20133 Italy; 2 Department of Psychology, University of Turin, Turin, Italy; 3 Department of Human and Social Sciences, University of Aosta Valley, Aosta, Italy; 4 Department of Biomedical and Clinical Sciences, Università di Milano, Milan, Italy; 5 Department of Territorial Activities, Azienda Sanitaria Provinciale, Health District, Catania, Italy; 6 Neurology Unit & Regional Referral Multiple Sclerosis Centre (CReSM), University Hospital San Luigi Gonzaga, Orbassano, Italy; 7 Department of Neuroscience, San Camillo-Forlanini Hospital, Rome, Italy; 8 Department of Neurosciences, Imaging and Clinical Sciences, University G. d’Annunzio, Chieti, Italy; 9 Psychological Service - Neurological and Neurological Rehabilitation Units, IRCCS San Raffaele, Milan, Italy; 10 Laboratory of Clinical Neuropsychology, Psychology Unit, ASST Lariana, Como, Italy; 11 IRCCS Don Gnocchi Foundation, Florence, Italy; 12 Multiple Sclerosis Center, Neurology Unit, Hospital of Vaio, Fidenza, Italy; 13 Azienda Sanitaria Locale, ASL-BA, Bari, Italy; 14 Multiple Sclerosis Center, Unit of Neuroimmunology and Neuromuscular Diseases, Fondazione IRRCS Istituto Neurologico Carlo Besta, Milan, Italy; 15 Department of Medical Science and Public Health, University of Cagliari, Cagliari, Italy; 16 Multiple Sclerosis Center, ASL Cagliari, ATS Sardegna, Cagliari, Italy; 17 Multiple Sclerosis Unit, IRCCS S. Lucia Foundation, Rome, Italy; 18 Dipartimento di Scienze Biomediche e Neuromotorie, Università di Bologna, Bologna, Italy; 19 IRCCS Istituto delle Scienze Neurologiche di Bologna, Bologna, Italy; 20 UOC Psicologia Ospedaliera, AUSL di Bologna, Bologna, Italy; 21 Department of Clinical Sciences and Translational Medicine, University of Rome “Tor Vergata”, Rome, Italy; 22 Behavioral Neuropsychology Laboratory, IRCCS S. Lucia Foundation, Rome, Italy; 23 Neurologia ad indirizzo Neuroimmunologico - Centro Sclerosi Multipla, Ospedale di Gallarate - ASST della Valle Olona, Gallarate, Italy; 24 Mathematics and Statistics, La Trobe University, Melbourne, Australia; 25 Neuroepidemiology Unit, Centre for Epidemiology and Biostatistics, Melbourne School of Population and Global Health, The University of Melbourne, Melbourne, Australia

**Keywords:** Multiple sclerosis, Bifactor model, Item response theory, Multidimensional computerized adaptive test, Health-related quality of life, MSQOL-54

## Abstract

**Background:**

The Multiple Sclerosis Quality of Life-54 (MSQOL-54) is one of the most commonly-used MS-specific health-related quality of life (HRQOL) measures. It is a multidimensional, MS-specific HRQOL inventory, which includes the generic SF-36 core items, supplemented with 18 MS-targeted items. Availability of an adaptive short version providing immediate item scoring may improve instrument usability and validity. However, multidimensional computerized adaptive testing (MCAT) has not been previously applied to MSQOL-54 items. We thus aimed to apply MCAT to the MSQOL-54 and assess its performance.

**Methods:**

Responses from a large international sample of 3669 MS patients were assessed. We calibrated 52 (of the 54) items using bifactor graded response model (10 group factors and one general HRQOL factor). Then, eight simulations were run with different termination criteria: standard errors (SE) for the general factor and group factors set to different values, and change in factor estimates from one item to the next set at < 0.01 for both the general and the group factors. Performance of the MCAT was assessed by the number of administered items, root mean square difference (RMSD), and correlation.

**Results:**

Eight items were removed due to local dependency. The simulation with SE set to 0.32 (general factor), and no SE thresholds (group factors) provided satisfactory performance: the median number of administered items was 24, RMSD was 0.32, and correlation was 0.94.

**Conclusions:**

Compared to the full-length MSQOL-54, the simulated MCAT required fewer items without losing precision for the general HRQOL factor. Further work is needed to add/integrate/revise MSQOL-54 items in order to make the calibration and MCAT performance efficient also on group factors, so that the MCAT version may be used in clinical practice and research.

## Introduction

Health researchers, clinicians, and policy makers are increasingly using patient-reported outcome measures (PROMs) to inform patient care and service provision, improve patient outcomes, and assess quality of care and performance indicators across healthcare organizations [1].

Health-Related Quality of Life (HRQOL) is one of the most used outcome measures in health research, clinical trials, and post-authorization studies [2]. It can be referred to as a ‘*subjective evaluation of the influence of health on the individuals’ ability of having a normal functioning which makes it possible to perform all the activities which are important for them and which affect their well-being’* [3, page 888]. While there is little agreement about which domains form the HRQOL construct, it is probably multifaceted or multidimensional [4]. Due to this multidimensionality, HRQOL instruments could potentially be very long, and burdensome for patients and clinicians.

The Multiple Sclerosis Quality of Life-54 (MSQOL-54) is one of the most widely used MS-specific HRQOL measures [5]. It is a multidimensional, MS-specific HRQOL inventory, which includes the generic SF-36 core items, supplemented with 18 MS-targeted items [6]. The instrument is well documented in terms of content [6], discrimination [6, 7], structural [6, 8], and cross-cultural validity [7–9], internal consistency [6, 7, 9], test-retest reliability [7], and responsiveness [10]. The questionnaire however has limitations including its length [11], a possible floor effect for the ‘physical function’ scale, and a high number of missing answers for ‘sexual function’ and ‘satisfaction with sexual function’ scales [7, 12, 13].

Computerized adaptive testing (CAT) could reduce patient and clinician burden [14] by shortening the questionnaire length, and could contribute to minimizing floor and ceiling effects by providing patients with individualized items. By connecting the item response theory (IRT) approach with strong computer capabilities, CAT represents a promising research area in QOL/PROM assessment. The starting point is typically an item bank including questions which are calibrated according to psychometric techniques [14, 15]. An item bank includes a large number of items with various difficulty levels covering different levels of a latent trait (in this case, HRQOL). For each individual, CAT administration starts with a first item selected from the item bank as the most informative for a given level of latent trait, typically the mean level. Based on each individual’s answer to the first item, an initial estimate of the latent trait score is made. Then, a second item is selected; its difficulty is based on the current estimation of the latent trait score. By responding to the second item, the latent trait score can be re-computed with higher precision. This procedure goes on, until a specific stopping rule is met (for example, a predetermined level of precision for latent trait estimate, a specified number of administered items).

Evidence shows that CAT has been used effectively in education, psychology [14, 16, 17], and healthcare settings [18–27].

By considering correlations between domains, multidimensional CAT (MCAT) may be a more efficient approach to assess HRQOL [28]. In MCAT, an item may provide information regarding one or more latent variables. Thus, items are chosen to maximize information across levels of latent traits over all the dimensions [14]. MCAT may use these associations to improve measurement efficiency. Paap et al., 2019 [29] found that MCAT was more efficient than unidimensional CAT in reducing test length and increasing precision, and this was true also in health measurements [30–32]. MCAT based on fixed-length questionnaires can be challenging as these questionnaires could be ‘too long to be used routinely, too short to ensure both content validity and reliability’ [33]. However, there is evidence of successful application of MCAT to fixed-length HRQOL questionnaires, including the MS domain [25, 34–36].

In a previous study conducted in MS [37], we found that a bifactor model fit the data well, suggesting that a unidimensional HRQOL score can be computed using the MSQOL-54. By definition, items in the bifactor model load on a general factor and on one group factor only. The general factor accounts for item correlation due to the broad construct of HRQOL; group factors account for item covariation that is independent of the covariation due to the general factor, and provide unique information on specific domains of HRQOL. Moreover, the general and group factors are uncorrelated [38].

The overall HRQOL score could be used in clinical practice to provide health professionals and MS patients with feedback on current functioning [37]. Also, it could be useful to identify patient subgroups—with different levels of disability as well as disease forms—in order to deliver personalized interventions addressing, for example, resilience or self-efficacy. Yet, for researchers, it could be easier to calculate and interpret a single HRQOL total score, when using such measure in clinical trials or other research studies [37].

MCAT approach based on a bifactor model could enable the implementation of an adaptive version of the questionnaire which may be particularly suited to the measurement of multidimensional HRQOL and its sub-domains, at the same time providing a single overall HRQOL score.

In the current study we aimed to apply MCAT to the MSQOL-54, and to investigate its performance in comparison to the full-length questionnaire, in terms of item reduction, and preservation of a precise score estimate.

## Methods

### Source of data

The data for the present secondary analysis are derived from different datasets collected with the English and Italian versions of the MSQOL-54 within ongoing or completed projects conducted in Australia and Italy [37, 39] (see Appendix, Additional File).

The dataset included 3669 MS patients (mean age 43.8 years [range 18–87], 74% women, 54% with a mild level of disability, and mean disease duration of 7.2 years [0–48]) (Table 1). Of these, 2064 (56%) were English- and 1605 (44%) were Italian-speaking [37, 39]. Data from the English and Italian versions were pooled after ensuring measurement invariance of the MSQOL-54 across the two language versions [39].

**Table 1:** Characteristics of the dataset (N = 3669 patients)

	Summary
Women, number (%)	2700 (74)
Mean age in years, SD (range)	43.8, 10.9 (18–87)
Mean years from MS diagnosis, SD (range)	7.2, 7.8 (0–48)
Median EDSS score (range)	2.5 (0–9.5)
PDDS (%)	
Mild disability	1110 (54)
Moderate disability	722 (35)
Severe disability	219 (11)
Mean MSQOL-54 PHC, SD (range)	59.2, 21.1 (1–100)
Mean MSQOL-54 MHC, SD (range)	65.0, 21.1 (1-100)

EDSS, Expanded Disability Status Scale; MSQOL-54, Multiple Sclerosis Quality of Life-54; PDDS, Patient Determined Disease Steps; PHC/MHC, Physical and Mental Health Composite; SD standard deviation

### Instrument

The MSQOL-54 comprises the generic Short-Form 36-item (SF-36) instrument [40], plus 18 MS-specific items derived from professionals’ advice and a literature review [6]. The 54 items have a mixed response format and are organized into 12 subscales plus two single items (Table 2) [6]. Item response format, administration forms, and scoring instructions are freely available here [41]. Items in all the subscales enquire about HRQOL over the preceding month, except for item 2 (change in health) which refers to the preceding year. Similarly to SF-36, two composite scores (Physical Health Composite, and Mental Health Composite) are derived by combining scores of the relevant subscales [6].

**Table 2:** MSQOL-54 subscales and items

Subscale	No. of items	Item number
Physical function	10	3–12
Role Limitations – Physical	4	13–16
Role Limitations – Emotional	3	17–19
Bodily pain	3	21, 22, 52
Emotional well-being	5	24–26, 28, 30
Energy/vitality	5	23, 27, 29, 31, 32
Health perceptions	5	1, 34–37
Social function	3	20, 33, 51
Cognitive function	4	42–45
Health distress	4	38–41
Social function	4	46–49
Change in health	1	2
Satisfaction with sexual function	1	50
Overall quality of life	2	53, 54

### Psychometric analysis

The secondary analysis conducted in the present study consisted of the following consecutive steps. First, we performed item calibration according to multidimensional item response theory (IRT) analysis using a bifactor model. Second, a series of simulations was conducted to apply MCAT to the MSQOL-54. Third, we assessed MCAT performance, in comparison to the full-length questionnaire.

#### Item calibration

As present data are ordinal in nature, we calibrated the item bank (i.e., the MSQOL-54 items) by using the bifactor IRT graded response model [42–44], which relates properties of the test items (e.g., discrimination and difficulty) to the latent trait of the subject. According to the bifactor factor structure of the MSQOL-54 reported in our previous publication [37], items 2 and 50 were not included in this model, because they are single-scale items.

Before item calibration, the local independence assumption of the items was evaluated by applying Yen’s Q3 index [45]. The Q3 index was calculated for every item pair (i,j) and corresponded to the correlation between item residuals after fitting the model. These item residuals were differences between the observed responses of the individual item and the response reproduced by the model. We considered item residual correlations above 0.20 to be indicative of local dependence between items [45]. We compared the information function for each item within pairs, and items with less information were accordingly removed from calibration and simulation analyses [46]. Missing data were treated by using a full information maximum likelihood method of estimation.

The goodness of fit of the bifactor IRT graded response model was evaluated with Root Mean Square Error of Approximation (RMSEA), Standardized Root Mean Square Residuals (SRMSR), and Comparative Fit Index (CFI) based on the limited information M2 statistic. According to the rule of thumb suggested by Maydeu-Olivares in 2013 [47], RMSEA and SRMR values ≤ 0.05 were deemed indicative of acceptable model fit. For CFI, the same fit criterion (≥ 0.95) employed in structural equation models was used because - to our knowledge - no systematic studies on CFI’s performance in the IRT framework are available in the literature. Local fit was assessed with the S-X2 statistic [48], after controlling for familywise Type I error rate [49] and using item-level RMSEA as a measure of effect size.

#### MCAT simulations

In line with recommendations made by Chalmers [50], we chose to conduct MCAT simulations using a randomly generated sample of 1000 respondents. According to the bifactor model which includes one general HRQOL factor and ten group factors, for each subject, 11 true latent traits (θs) were generated from a multivariate normal (MVN) distribution with MVN (0, 1) [51], and with no correlations among θ_S_ [37] and simulated item responses to all 44 items were obtained using the item parameters from the calibration step. The θ_s_ were generated with the *mvtnorm* package in R [52] (version 3.4.3).

In line with Sunderland et al. [17], we ran a simulation study using the following three termination criteria: (a) standard errors (SE) for the general HRQOL factor; (b) SE for group factors; and (c) change in *θ* estimates ($\widehat{\theta }$) from one item to the next for both the general and the group factors (Table 3). For each criterion, two levels were considered, obtaining a 2 × 2 × 2 design described in Table 3. A (‘full’) simulation with no termination rules was conducted to generate a comparison instrument in which all items were administered adaptively.

**Table 3:** Simulations design

	SE on general factor	SE on group factor										Change in $\widehat{\theta }$ from one item to the next for both the general and the group factors
Simulation	HRQOL	Physical function	Role limitations-physical	Role limitations-emotional	Bodily pain	Emotional well-being	Energy/vitality	Health perceptions	Cognitive function	Health distress	Sexual function	
Full*	-	-	-	-	-	-	-	-	-	-	-	-
1	0.32	0.50	0.50	0.50	0.50	0.50	0.50	0.50	0.50	0.50	0.50	-
2	0.32	0.50	0.50	0.50	0.50	0.50	0.50	0.50	0.50	0.50	0.50	< 0.01
3	0.32	-	-	-	-	-	-	-	-	-	-	-
4	0.32	-	-	-	-	-	-	-	-	-	-	< 0.01
5	0.40	0.50	0.50	0.50	0.50	0.50	0.50	0.50	0.50	0.50	0.50	-
6	0.40	0.50	0.50	0.50	0.50	0.50	0.50	0.50	0.50	0.50	0.50	< 0.01
7	0.40	-	-	-	-	-	-	-	-	-	-	-
8	0.40	-	-	-	-	-	-	-	-	-	-	< 0.01

HRQOL, health-related quality of life; SE, standard error of measurement; $\widehat{\theta }$, latent trait estimate

* Simulation conducted with no termination rules

As shown in Table 3, for the general HRQOL factor, SE was set to 0.32 (simulations 1–4) and 0.40 (simulations 5–8). We chose these values as they correspond to reliability values of 0.90 and 0.85, respectively (calculated with the formula: reliability = 1- (SE^2^) [53]), which are generally required for minimal reliability in individual assessments [54]. In addition, these thresholds were employed in other studies in the HRQOL field [55, 56].

For group factors, we used SE set to 0.50 - corresponding to a reliability value of 0.75- (simulations 1–2, 5–6), and ‘no SE threshold’ (simulations 3–4, 7–8), to take into account the multidimensionality of the MSQOL-54, and that the group factors included a small number of items.

For the third criterion, i.e., the change in $\widehat{\theta }$, we used a threshold of < 0.01 for both the general and the group factors (simulations 2, 4, 6, and 8) and ‘no threshold’ (simulations 1, 3, 5, and 7). We chose this threshold value as described by Sunderland et al. [17] because it provided an optimal balance between efficiency and precision.

In simulations 1, 2, 5, and 6 (Table 3), the SE rules associated with the general factor and the 10 group factors were applied simultaneously: MCAT would terminate if both the SE associated with the estimates for the general HRQOL factor and each of the group factor dropped below the threshold. In simulations 2, 4, 6 and 8, MCAT would terminate if one of the two criteria (SE rule for all the factors involved and changes in ${\widehat{ \theta }}_{s}$) was fulfilled.

For each MCAT, the most informative item, for each individual with an average latent trait level, was used as the starting item. To select the starting item, the DP-rule was used, which consists in calculating the determinant of the posterior information matrix for each item in the item bank, and selecting the item for which the highest value is given [57]. The same criterion was used to select the subsequent items, considering the answers to previously administered items. We chose this criterion, as it improves the estimation of the general HRQOL factor scores [58].

Latent trait estimates for the general and group factors were obtained via the multidimensional maximum a posteriori (MAP) estimator [42]. We chose the MAP estimator rather than the expected a posteriori (EAP) or maximum likelihood (ML) estimator, because the MAP estimator provides better precision than maximum likelihood when the a-priori distribution corresponds to the latent distribution – as it is in our study based on simulated multinormal latent traits – and performs as well as the EAP estimator with the advantage of a lower computational burden than EAP [58]. Moreover, the MAP estimator was used in similar studies applying MCAT using bifactor modeling [17, 59].

#### MCAT performance

Performance of the MCAT was assessed by calculating the root mean square difference (RMSD), and the mean, median number (interquartile range, IQR) of items administered, and item reduction as compared to the full-length questionnaire.

RMSD was determined by comparing MCAT latent trait estimates with simulated true latent traits. RMSD was calculated as follows:

$$RMSD= \sqrt{\frac{{\sum }_{J=1}^{N}({\widehat{\theta }}_{j}-{\theta }_{j}{)}^{2}}{N}}$$

Here, ${\widehat{\theta }}_{j}$ represents estimated latent trait level for the *j*^th^ examinee for each research condition tested, ${\theta }_{j}$ indicates the true latent trait value for each examinee, as defined above, and *N* is the number of examinees [60, 61]. A low RMSD value indicates a more accurate measurement [17, 62].

We also calculated Pearson’s correlations to compare $\widehat{\theta }$ for each MCAT simulation with the true latent trait values.

### Software

Analyses were performed using R (version 3.4.3) [52]. We modeled the responses to the MSQOL-54 items using the bifactor IRT model with the *mirt* package [63], and for the MCAT simulations we used the *mirtCAT* package [50].

## Results

### Item calibration

Before item calibration, we assessed whether the 52 items met the assumption of local independence. Such local dependency (i.e., residual correlations > 0.20) apparently was between ten item pairs: 5 and 10, 30 and 54, 9 and 10, 6 and 7, 4 and 5, 10 and 11, 44 and 45, 20 and 33, 29 and 31, 53 and 54 (see Supplementary Tables 1, Additional File).

Items 30 and 54 had similar content, as well as items 20 and 33, and items 53 and 54. Further, items 29 and 31 had similar stem. Items 4 and 5 had similar content and were presented sequentially, as well as items 44 and 45, and items 53 and 54. Finally, items 9 and 10 had similar stem, content, and were presented sequentially, as well as items 6 and 7; 10 and 11.

Thus, by further inspecting the item information function within pairs, we removed eight items having the lower information function from the subsequent MCAT simulations (i.e., 5, 6, 9, 11, 20, 29, 45, 54) (see Supplementary Table 1, Additional File).

The bifactor IRT graded response model showed quite good fit on the resulting 44 items; particularly, RMSEA and CFI satisfied the fit criteria (RMSEA = 0.047; CFI = 0.980) and only SRMSR was lightly above the threshold value (SRMSR = 0.061). By examining the fit at item level, 6 of the 44 items resulted as misfitting at p < 0.05 after Benjamini–Hochberg correction for Type I error rates (see Supplementary Table 2, Additional File). However, the corresponding RMSEA values were small (max RMSEA = 0.02), indicating negligible deviation of the items from the bifactor graded response model.

As shown in Supplementary Table 2 included in the Additional File, the item discrimination values were high for almost all the items on both the general factor (ranging from 0.92 to 4.71) and the group factors (ranging from 0.56 to 5.19); the few parameters below the value of 1 were those of items 24, 34 and 36 on the general factor and those of items 31, 32, 34 and 36 in the group factor. Items’ difficulty/thresholds parameters were widely spread across the latent continuum.

**Fig. 1:**
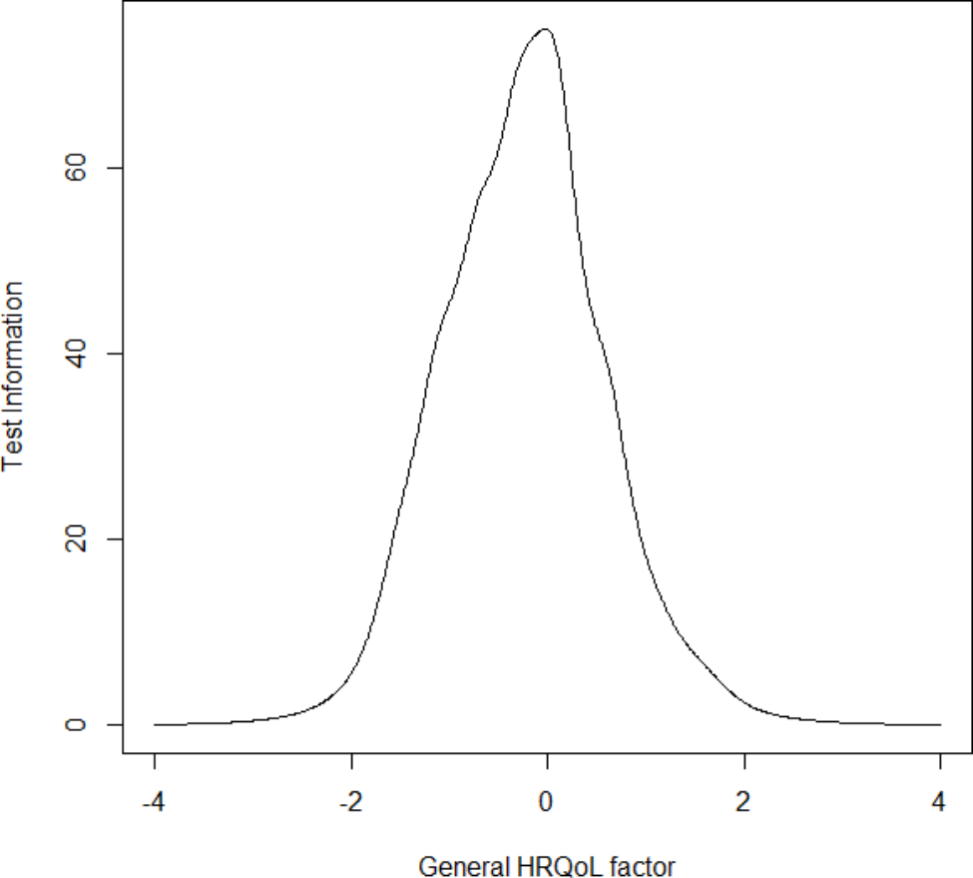
Test information curve of the general health-related quality of life (HRQOL) factor

Figure 1 shows the information distribution [63] for the general HRQOL factor (higher scores correspond to higher quality of life), suggesting that most HRQOL information is provided in a range around − 2 to 2, with maximum information around zero.

### MCAT simulations

The matrix of item parameter estimates from the bifactor graded response model calibration including 44 items, and the matrix of simulated item responses derived from the MVN distribution were processed.

The (‘full’) solution including all the 44 items showed that the mean SE on the general HRQOL factor was 0.28, the correlation of $\widehat{\theta }$ with *θ* was 0.96, and RMSD was 0.29 (Table 4).

**Table 4:** MCAT performance measures and item reduction on general HRQOL factor for each simulation

Simulation	Mean number of administered items, median (interquartile range)	Item reduction	Mean SE (range)	Correlation between $\widehat{\theta }$ and *θ*	RMSD
Full	44	-	0.28 (0.26–0.43)	0.96	0.29
1	44	0	0.28 (0.26–0.43)	0.96	0.29
2	31, 35 (21–42)	28.7%	0.31 (0.26–0.55)	0.94	0.32
3	26, 24 (22–29)	41.1%	0.31 (0.29–0.43)	0.94	0.32
4	23, 22 (20–27)	48.5%	0.33 (0.29–0.55)	0.94	0.34
5	44	0	0.28 (0.26–0.43)	0.96	0.29
6	31, 35 (21–42)	28.7%	0.31 (0.26–0.55)	0.94	0.32
7	10, 9 (9–10)	77.6%	0.39 (0.36–0.43)	0.91	0.41
8	10, 9 (9–10)	78%	0.39 (0.36–0.55)	0.91	0.41

HRQOL, health-related quality of life. MCAT, multidimensional computerized adaptive testing. RMSD, root mean square difference﻿. SE, standard error of measurement. $\widehat{\theta }$, latent trait estimate. *θ*, true latent trait

Among the eight implemented simulations, two pairs (i.e., 1, 5 and 2, 6; Table 4) provided the same results. In both cases, the simulation design differed only regarding the SE value for the general factor (0.32 and 0.40, respectively). In detail, simulations 1 and 5 - in which SE for group factors was set to 0.50 and the change in $\widehat{\theta }$ was not used as a stopping rule - led to the administration of all items. In simulations 2 and 6 (that respectively differed from 1 to 5 for the presence of the stopping rule related to the change in $\widehat{\theta }$ from one item to the next), the median number of administered items was 35 (IQR 21–42), RSMD was 0.32, and the correlation with *θ* was 0.94 (Table 4).

Because both simulations 1 and 5 and simulations 2 and 6 led to the same results, only simulations 1 and 2 were considered thereafter, knowing that results held also for simulations 5 and 6.

In simulation 3 (i.e., SE set to 0.32 on the general factor, and no thresholds for group factors), the median number of administered items was 24 (IQR 22–29), representing a 41% reduction in respondent burden, RMSD was 0.32, and the correlation with *θ* was 0.94. For simulation 4 (i.e., SE set to 0.32 on the general factor, no SE thresholds for group factors, and change in $\widehat{\theta }$ from one item to the next), the median number of administered items was 22 (IQR 20–27), RMSD was 0.34, and correlation was 0.94.

For simulation 7 (i.e., SE set to 0.40 on the general factor, and no thresholds for group factors) and simulation 8 (same criteria as the previous simulation plus change in $\widehat{\theta }$ from one item to the next), the median number of administered items was 9 (IQR 9–10), RMSD was 0.41, and correlation was 0.91 for both simulations, resulting in 78% of item reduction.

Compared to the other simulation, simulation 3 showed the best compromise between item reduction and general factor correlation between $\widehat{\theta }$ and *θ.* It led to a 41% item reduction, preserving a high correlation with *θ* (0.94). This satisfactory performance was further supported by the results of the comparative performance in terms of gain/loss of each measure (i.e., SE, RMSD, and correlations for general and group factors) for each simulation, in comparison to the (full) simulation where all items were administered (see Supplementary Fig. 1, Additional File).

Regarding the group factors, although simulation 3 was the best according to the above-mentioned gain and loss results, its performance was only marginally satisfactory: On average the mean SE was 0.55, the mean correlation of $\widehat{\theta }$with *θ* was 0.80, and mean RMSD was 0.58 (see Supplementary Table 3, Additional File). This is due to the small number of items for each group factor; in fact, also the “full” solution including all the 44 items showed satisfactory, but not excellent results (on average the mean SE was 0.51, the mean correlation of $\widehat{\theta }$with *θ* was 0.84, and mean RMSD was 0.54).

**Fig. 2:**
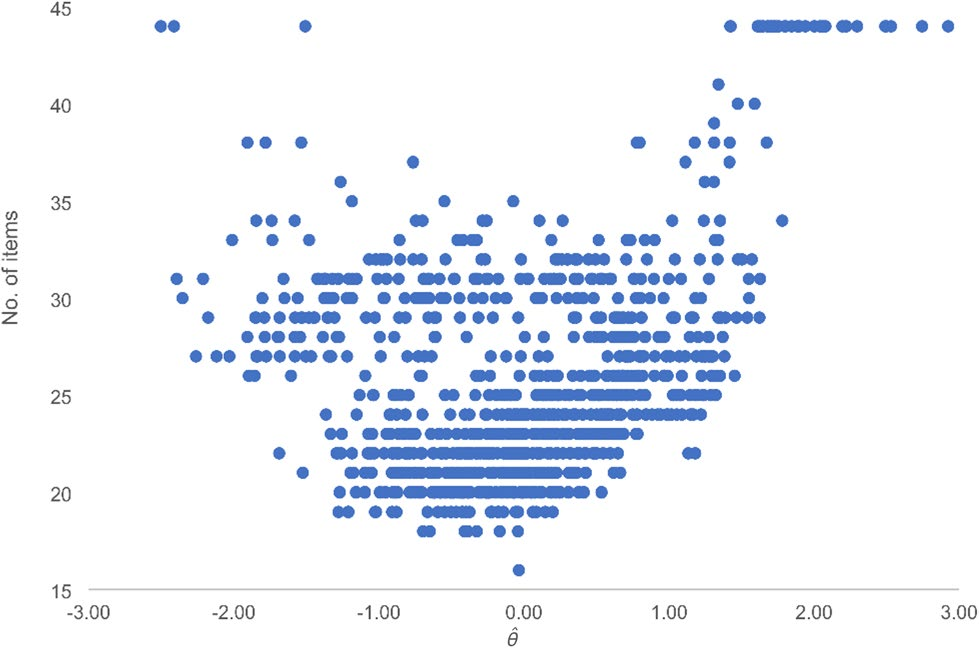
Relationship between number of items administered and level of health-related quality of life ($\widehat{\theta }$) in simulation 3

Figure 2 presents the relationship between number of administered items and level of HRQOL in simulation 3. Here, the number of items used in MCAT was lowest for patients whose underlying level of the measured construct (i.e., HRQOL) was between ± 2 logits, and highest for those at the extreme ends of the spectra (± 3 logits). Supplementary Fig. 2 included in the Additional File reports the relationship between number of items administered and level of HRQOL in the other simulations performed in the study.

## Discussion

In the present study, we ran eight simulations, and evaluated MCAT performance for the MSQOL-54. Findings from MCAT simulations indicated that the simulation with SE set to 0.32 on the general HRQOL factor, no SE thresholds on group factors and no application of the Sunderland et al. criterion among the stopping rules, outperformed the other simulations, and provided satisfactory performance.

The simulations using changes in $\widehat{\theta }$ as additional stopping rules resulted in significant item reduction in two cases (48.5% and 78%). Nevertheless, they did not achieve satisfactory performance measures.

As far as we know, this is the first attempt to apply MCAT to the MSQOL-54. Research in this field is sparse. There are a few examples in the literature [25, 34–36] reporting results using other instruments, such as the MusiQOL [36], but none of these studies used an MCAT approach based on a bifactor IRT model.

Our study has some limitations. First, we performed MCAT simulations using a fixed-length questionnaire with a relatively short item pool not specifically developed for computerized adaptive testing. With respect to questionnaire length, an item bank should be large enough to provide adequate precision over the full range of the latent constructs. Here, 44/54 (81%) of the original items were calibrated and used in the simulations. This is a relevant limitation in that such 44-item multidimensional item pool with several subscales may have limited the performance of the simulations, with the risk of ending up with one item per subscale.

Further, the MSQOL-54 was developed in 1995, and it was suggested that researchers should perform item ‘seeding’ at a certain time to maintain and renew item banks [14]. To overcome this issue, further work should be conducted to add/integrate/revise items of the MSQOL-54, in order to make the calibration and MCAT performance more efficient on group factors.

Another limitation is that we preferred to use a matrix of simulated item responses in the MCAT simulations. A few drawbacks of these simulations should be acknowledged. Specifically, they are time-consuming to perform, and their outcomes derive from an unlikely situation in which data totally fit the model. Importantly, considering that it is a preliminary study, our results should be generalized with caution to other MS patient groups, as occurs in real-data simulations where *θs* are not obtainable [64].

Based on our findings, a number of further steps are warranted. After working on adding/integrating/revising items of the MSQOL-54, validation studies using an independent MS sample could be prospectively conducted, including other socio-demographic and clinical variables (e.g., education, employment, and disease course), as well as other relevant PROMs. This could be done in order to further explore MCAT performance, and the external validity of the adaptive version. The same validation studies could be conducted using a longitudinal design, so as to assess over time other important psychometric properties, such as sensitivity to change or test-retest reliability. In these studies, a testing platform could be used to deploy MCAT to the patients, using also mobile devices.

Despite study limitations, present results have important implications for clinical practice and research. The MCAT approach can provide patients, clinicians, and researchers with immediate feedback, by reducing item numbers and tailoring items to the individual patient, thus improving the efficiency and precision of the instrument. This can increase accuracy, make the instrument interpretable, and shorten the time spent for questionnaire administration, thus reducing patient burden. In our selected simulation, a reduction of 41% of administered items was reported. This could have a significant impact on clinical practice, where time is at a premium. Though preliminary, these results could also have an impact on the patient-physician relationship and shared decision making as, incorporating patient perspectives is crucial to improve care outcomes and is a key component of patient-centered care [65].

The MCAT version of the MSQOL-54 could potentially be employed also at the group level data; it could be integrated in the electronic health records, as well as in MS registries, both at the national [66] and international levels [67–70]. Further, another novel method to incorporate such an MCAT version of the MSQOL-54 into practice could be patient portals. These portals are generally linked to electronic health records, allowing patients to monitor their health [71]. With the objective of making the information immediately available to patients, such portals may represent the next step to further integrate PROMs into clinical practice, thus improving quality of care.

## Conclusion

This research was part of an ongoing international collaborative project between Italian and Australian investigators. It provided promising evidence that an MCAT version of the MSQOL-54 could be developed in the future; further work is needed to add/integrate/revise the original MSQOL-54 item pool. Then, the adaptive instrument could be used in clinical practice and research providing notable item reduction and decreasing patient and clinician burden, while preserving high accuracy levels.

## Data Availability

The dataset generated and analyzed during the current study is available in the Zenodo repository, 10.5281/zenodo.4591136.

## Declarations

### Supplementary information

Additional file: Appendix. Source of data of calibration sample. Supplementary Table 1. Item residual correlations and items which were removed. Supplementary Table 2. MSQOL-54 item parameter estimates and misfit. Supplementary Fig. 1. Comparative performance in terms of gain/loss of each measure for each simulation, in comparison to the simulation where all items were administered. Supplementary Table 3. MCAT summary performance measures (mean, min-max) on group factors for each simulation. Supplementary Fig. 2. Relationship between number of items administered and level of health-related quality of life in other simulations performed in the study.
